# E series prostaglandins alter the proliferative, apoptotic and migratory properties of T98G human glioma cells in vitro

**DOI:** 10.1186/1476-511X-11-171

**Published:** 2012-12-11

**Authors:** Renata N Gomes, Alison Colquhoun

**Affiliations:** 1 Department of Cell and Developmental Biology, University of São Paulo, São Paulo, CEP 05508-900, SP, Brazil

**Keywords:** Glioma, Prostaglandin, Ibuprofen, Apoptosis, Migration

## Abstract

**Background:**

In many types of cancer, prostaglandin E_2_ (PGE_2_) is associated with tumour related processes including proliferation, migration, angiogenesis and apoptosis. However in gliomas the role of this prostanoid is poorly understood. Here, we report on the proliferative, migratory, and apoptotic effects of PGE_1_, PGE_2_ and Ibuprofen (IBP) observed in the T98G human glioma cell line in vitro.

**Methods:**

T98G human glioma cells were treated with IBP, PGE_1_ or PGE_2_ at varying concentrations for 24–72 hours. Cell proliferation, mitotic index and apoptotic index were determined for each treatment. Caspase-9 and caspase-3 activity was measured using fluorescent probes in live cells (FITC-LEHD-FMK and FITC-DEVD-FMK respectively). The migratory capacity of the cells was quantified using a scratch migration assay and a transwell migration assay.

**Results:**

A significant decrease was seen in cell number (54%) in the presence of 50 μM IBP. Mitotic index and bromodeoxyuridine (BrdU) incorporation were also decreased 57% and 65%, respectively, by IBP. The apoptotic index was increased (167%) and the in situ activity of caspase-9 and caspase-3 was evident in IBP treated cells. The inhibition of COX activity by IBP also caused a significant inhibition of cell migration in the monolayer scratch assay (74%) and the transwell migration assay (36%).

In contrast, the presence of exogenous PGE_1_ or PGE_2_ caused significant increases in cell number (37% PGE_1_ and 45% PGE_2_). When mitotic index was measured no change was found for either PG treatment. However, the BrdU incorporation rate was significantly increased by PGE_1_ (62%) and to a greater extent by PGE_2_ (100%). The apoptotic index was unchanged by exogenous PGs. The addition of exogenous PGs caused an increase in cell migration in the monolayer scratch assay (43% PGE_1_ and 44% PGE_2_) and the transwell migration assay (28% PGE_1_ and 68% PGE_2_).

**Conclusions:**

The present study demonstrated that treatments which alter PGE_1_ and PGE_2_ metabolism influence the proliferative and apoptotic indices of T98G glioma cells. The migratory capacity of the cells was also significantly affected by the change in prostaglandin metabolism. Modifying PG metabolism remains an interesting target for future studies in gliomas.

## Background

Malignant gliomas and especially Glioblastoma multiforme (GBM) are the most malignant and prevalent intracranial tumours, classified as grade IV by the World Health Organization (WHO). GBMs are characterized by genetic alterations affecting genes that control cell growth, migration, apoptosis, and invasion. Despite very aggressive treatment including surgery and combined radio and chemotherapy the median survival for most patients with GBM is only 1 year. Therefore there is an urgent need for the development of novel therapeutic agents [1,2].

Non-steroidal anti-inflammatory drugs (NSAIDs) are widely used in the treatment of pain, fever and inflammation caused by various physiological or pathological conditions. Clinical trials have demonstrated that long-term NSAID use significantly reduces the risk of colorectal cancer and other tumours such as breast, lung, prostate and gastric cancer [3-5].

NSAIDs are known to inhibit a variety of cellular processes including signal transduction, transcription, and DNA repair. NSAIDs can alter cell cycle distribution, inhibit cyclins, modulate Bcl-2 family proteins and induce apoptosis [6,7]. NSAIDs also inhibit angiogenesis, an important factor necessary for tumour growth and survival, suggesting a rationale for their potential therapeutic application as anticancer agents [8].

The mechanism by which NSAIDs exert their anti-inflammatory activity is primarily by inhibiting the synthesis of prostaglandins through inhibition of both cyclooxygenase isoforms (COX-1 and COX-2), the rate-limiting enzyme of the cascade. COX-1 is constitutively expressed in many tissues and plays an important role in the control of homeostasis. Conversely, COX-2 is an inducible enzyme and is activated in response to extracellular stimuli such as growth factors and pro-inflammatory cytokines [9,10].

Several studies have shown COX and PGs play a role in cell growth, survival, migration/invasion and angiogenesis of tumour cells. Accumulating evidence suggests that the increase in overexpression of COX-2 and PGE_2_ in human glioma is associated with poor prognosis and tumour progression [11,12].

Ibuprofen (IBP) belongs to the group of NSAIDs, and is a potent COX-1 and COX-2 inhibitor. Besides its widespread use in the treatment of inflammatory diseases, it has been shown that IBP may be effective in the treatment and/or prevention of cancers including prostate and colorectal cancer [13-15].

However, the effect of IBP treatment in GBM has not been widely investigated. Recent studies from our laboratory have shown novel ruthenium-containing IBP complexes have significant effects on glioma cell proliferation and apoptosis [16,17]. In this study, we aimed to assess the potential effects of IBP on tumour cell proliferation, migration and apoptosis in T98G human glioma cells. The effect of the addition of exogenous PGE_1_ and PGE_2_ to the cells was also studied. PGE_1_ was compared with PGE_2_ as it has been reported to interact not only with the EP receptors EP3 and EP4 but also with the IP receptor, while PGE_2_ can interact with all four EP receptors. The cellular response to PGE_1_ and PGE_2_ depends on both the expression of receptors and the synthetic capacity of the individual tissue [18].

The study aimed to test the importance of PGE_1_ and PGE_2_ metabolism to the proliferative and apoptotic indices of T98G glioma cells. Since prostanoids are also involved in cell migration the effect of IBP, PGE_1_ and PGE_2_ on the migratory capacity of the cells was investigated using two migration assays.

## Methods

### Cell culture

T98G cells, derived from a human glioblastoma, were obtained from the ATCC and donated by Prof. S.S. Maria-Engler. Cells were grown in Dulbecco’s modified Eagle’s medium (DMEM) supplemented with 10% fetal calf serum (FCS), 50U/ml penicillin, and 50 μg/ml streptomycin. Cells in the exponential phase of growth were used, growing in 75 cm^2^ flasks in a humidified atmosphere of 5% CO_2_, 95% air at 37°C. After growth to the desired density cells were washed with PBS and trypsinized (trypsin 0.025%/ EDTA 0.02%) for subsequent plating. All experiments used cells grown in DMEM with FCS and antibiotics as stated above.

### Drug treatment

Ibuprofen (Cayman Chemicals) was dissolved in ethanol to achieve the stock concentrations desired. PGE_1_ and PGE_2_ (Cayman Chemicals) were dissolved in dimethyl sulfoxide (DMSO; Sigma-Aldrich). All drugs were further diluted in DMEM (Gibco BRL) to their final concentration.

### Cell proliferation assay

The cells were treated with IBP (25–200 μM), PGE_1_ (0.01–10 μM) or PGE_2_ (0.01–10 μM) for up to 72 hours in 75 cm^2^ flasks. After that, the cells were harvested with trypsin (trypsin 0.025%/ EDTA 0.02%) and the cell number was determined in an improved Neubauer counting chamber [19]. The cell proliferation assay was conducted three times for each concentration.

### Analysis of nuclear morphology

The IBP, PGE_1_ or PGE_2_ treated and control cells were fixed in situ with 4% formaldehyde in 0.1 M potassium phosphate buffer at pH 7.2. The cells were washed (x3) with PBS, pH 7.2, and incubated with 5 μg/ml Hoechst 33342 for 5 min at room temperature. The cells were examined and immediately photographed using a fluorescence microscope (Nikon Optiphot-II epifluorescence microscope equipped with, a Cool Snap Pro camera, and Image Pro plus software). Apoptotic cells can be distinguished from viable cells by their nuclear morphology with nuclear condensation and fragmentation, as well as by the higher intensity of blue fluorescence of the nuclei. The mitotic index was determined by the number of cells in cell division. A total of 200 cells were counted in multiple randomly selected fields, and the percentage of apoptotic and mitotic index cells were then calculated per total cell number.

### Migration assay

Cells were plated into 30 mm petri dishes (1.5 x 10^5^/dish) and maintained in a humidified atmosphere of 5% CO_2_: 95% air at 37°C. During the given period of 48 hours, the cells were treated daily with IBP (25 μM and 50 μM), PGE_1_ (10 μM) or PGE_2_ (10 μM). Before the end of treatment, the cell layer was scraped with a sterile razor blade (marking the point of zero migration) and the petri dish returned to the incubator for a further 10 hours in the presence of treatments. At the end of this 10 hours incubation period, the cells were fixed in situ with 4% formaldehyde in 0.1 M potassium phosphate buffer at pH 7.2. The cells were washed (x3) with PBS, pH 7.2, and incubated with 5 μg/ml Hoechst 33342 for 5 min at room temperature. The samples were washed (x3) with PBS, pH 7.2, before mounting in SlowFade mounting medium. Cells were analyzed with a Nikon Optiphot-II epifluorescence microscope equipped with, a Cool Snap Pro camera, and Image Pro plus software. The results were expressed as the number of migrating cells per mm^2^ of scratched area of petri dish [20].

### Transwell migration assay

A second migration assay was performed using the 24-well Boyden chamber plate with 8-μm pore size polycarbonate membrane filters. T98G cells (5 × 10^4^/well) were placed in the upper part of the Boyden chamber containing DMEM and 10% FCS, the lower chamber also contained DMEM and 10%FCS. After a period of cell adhesion (12 h) the medium was changed and treatment (IBP, PGE_1_ and PGE_2_) was added to both parts of the chamber. The culture media with treatment was changed daily for 48 h. After incubation, the cells on the membrane filter were fixed with methanol and stained with 0.05% crystal violet for 30 min.

The cells on the upper surface of the filter were removed with a cotton swab. The membranes were then rinsed in PBS until excess stain was removed. The membranes were then were air-dried for 20 min. The migratory cells were determined by counting the cells that migrated to the lower side of the filter by bright field microscopy at 200× magnification. Five random fields were counted for each filter, and each sample was assayed in triplicate.

### 5-Bromodeoxyuridine incorporation (BrdU)

BrdU incorporation assay (5-Bromodeoxyuridine incorporation) was used to evaluate the synthesis of DNA. T98G cells were seeded in 6-well plates (5 × 10^4^/well), followed by treatments for 72 h, as indicated. Cells were then incubated with 10 μM BrdU from 45 min, followed by fixation in 4% formaldehyde. After this, cells were washed with PBS/ 0.2% Triton and incubated with 2 M HCl for 10 min at room temperature. At the end of 10 min, the cells were placed in an oven at 40°C for 20 min. The blocking of endogenous peroxidase activity was carried out for 30 min with H_2_O_2_ (3%) in methanol/H_2_O followed by washing. The blocking of nonspecific sites was carried out with glycine and normal donkey serum for 1 h. The cells were then incubated “overnight” with primary anti-BrdU antibody. On the following day cells were incubated for 90 minutes with biotinylated secondary antibody and then washed again with PBS/0.2% Triton. The secondary antibody was detected by incubation with a streptavidin-biotinylated horseradish peroxidase for 60 min and washed, before reaction with 3,3′- diaminobenzidine (DAB)/H_2_O_2_. Cells were left in the developing solution for 2–10 min, counter-stained with 0.1% methyl green, dehydrated and mounted with Permount. The samples were photographed at random in five different fields and the percentage of BrdU positive cells was determined as a percentage of the total cell population [21].

### Caspase-9 and Caspase-3 activity assay

T98G cells were seeded in 12 well plates (5 × 10^4^/well) and incubated for 72 h with IBP, PGE_1_ or PGE_2_. After this period the cells were harvested with trypsin and centrifuged at 1500 rpm for 2 min. The pellet was resuspended in culture medium with treatment, 1 μl FITC-LEHD-FMK substrate for caspase-3 or 1 μl FITC-DEVD-FMK substrate for caspase-9, and 1 μl Hoechst 33342 at 37°C. Cells were washed and resuspended in caspase assay kit buffer. After mounting, the cells were viewed and photographed in a fluorescence microscope Nikon Optiphot-II equipped with a Cool Snap Pro camera and Image Pro Plus software.

### Statistical analysis

All data are presented as the mean ± SEM. Statistical analysis was carried out with the Graphpad InStat software. One-way ANOVA with a multiple comparison *t*-test was used for data analysis. Difference at level of p < 0.05 was considered to be significant.

## Results

### Effect of IBP on cell proliferation and apoptosis

**Figure 1:**
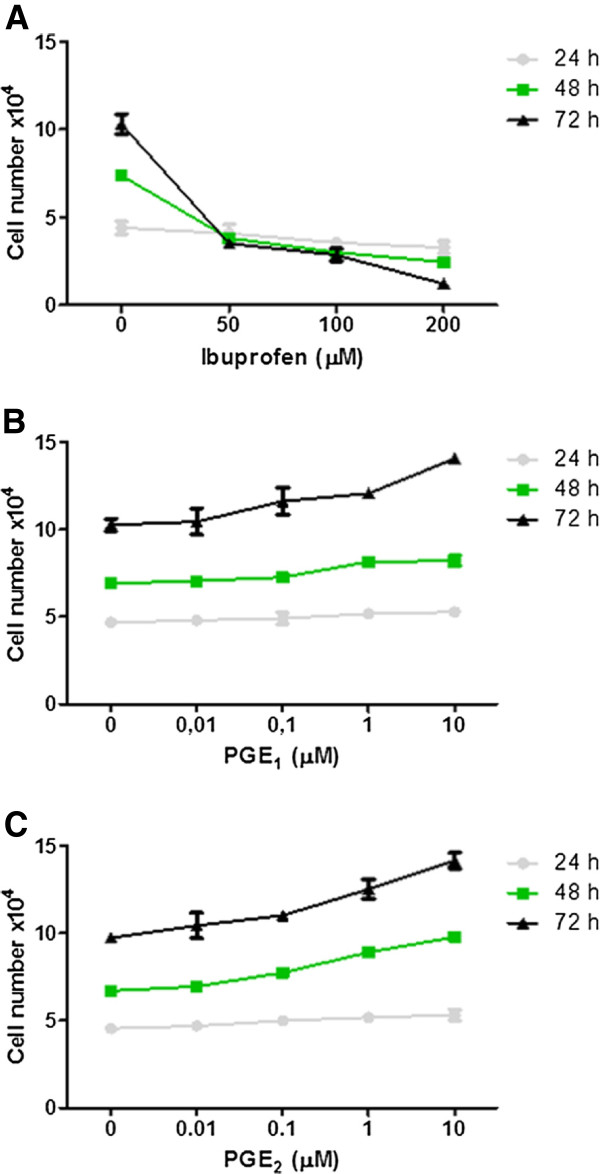
**Effect of IBP, PGE**_**1**_**and PGE**_**2**_**on T98G cell number.** (**A**) Cell number decreased dose and time dependently in the presence of IBP. (**B**) and (**C**) Cell number increased dose and time dependently in the presence of PGE_1_ and PGE_2_. Data are from three independent experiments in triplicate. Statistical significance, * p < 0.05 vs control. These experiments were designed to determine the concentrations and times of incubation for each compound for the remainder of the study.

To determine the importance of PGE_1_ and PGE_2_ to cell proliferation, T98G cells were treated with IBP, PGE_1_ or PGE_2_ for 72 h (Figure 1). Cell counting showed that the cell number was time and dose dependently decreased by IBP (Figure 1A). In contrast, the presence of PGE_1_ or PGE_2_ caused a time and dose dependent increase in cell number (Figure 1B and 1C). Further experiments in the study used the concentrations of 25 and 50 μM IBP, 10 μM PGE_1_ and 10 μM PGE_2_.

**Figure 2:**
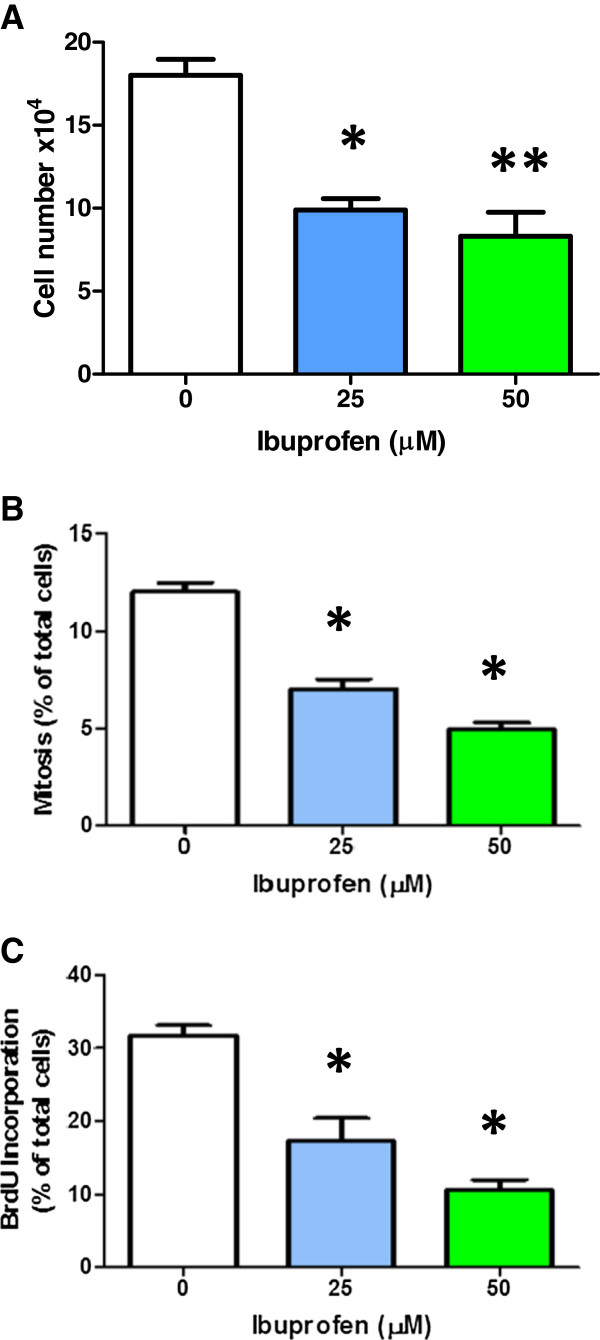
**Effect of IBP on cell number, mitotic index and bromodeoxyuridine (BrdU) incorporation in T98G cells.** (**A**) IBP decreased cell number after 72 h at both 25 and 50 μM. Statistical significance, *p = 0.0010 and **p = 0.0005, n = 3. (**B**) The number of cells in mitosis was obtained by Hoechst 33342 staining, and counting at x200 magnification. Statistical significance, *p = 0.0001, n = 8. (**C**) The nuclear incorporation of BrdU in the S-phase was measured after DAB staining and methyl green counterstaining. Statistical significance, *p < 0.0011, n = 4. Each experiment was repeated at least three times.

Cell counting showed that treatment with the lower dose of IBP inhibited proliferation by 45% and at 50 μM this inhibition increased to 54% (Figure 2A).

**Figure 3:**
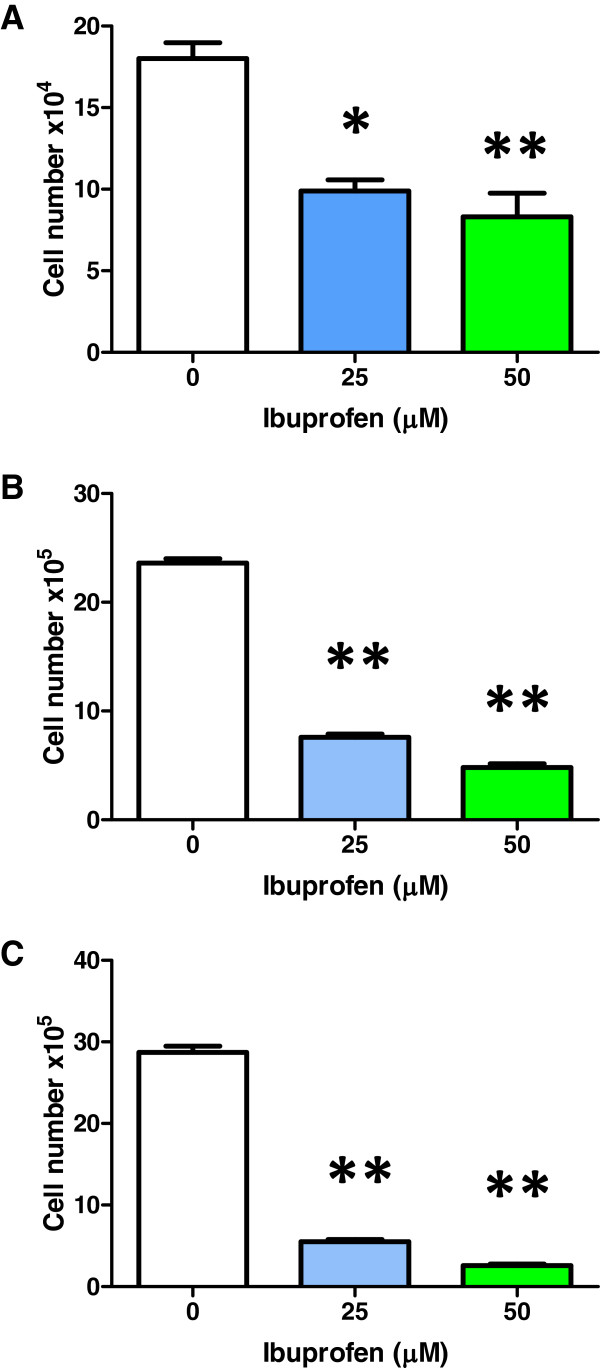
**Effect of IBP after 3, 5 and 10 days on T98G cell number.** (**A**-**C**) IBP decreased cell number after 3, 5 and 10 days at both 25 and 50 μM. Statistical significance, *p = 0.0010 and **p = 0.0005, n = 3.

Additional experiments proved that longer periods of exposure to IBP caused an even greater decrease in cell number (Figure 3). Cell numbers at 5 days IBP were decreased by 67.8% and 79.7% for 25 μM and 50 μM, respectively. After 10 days IBP cell number were decreased by 80.8% and 91.0% for 25 μM and 50 μM, respectively. After 10 days IBP at 50 μM the decrease in cell number (91.0%) reached levels greater than 3 days IBP at 200 μM (88.4%) (Figure 1A).

The fraction of cells in mitosis was quantified by calculation of the mitotic index. The fraction of cells in mitosis also decreased by 57% at 50 μM IBP (Figure 2B). Similarly, the percentage of BrdU positive cells decreased by 50% (25 μM IBP) and 65% (50 μM IBP) (Figure 2C). The overall finding for cell proliferation was an approximately 55–60% decrease in the presence of 50 μM IBP by the three methods used in the study.

**Figure 4:**
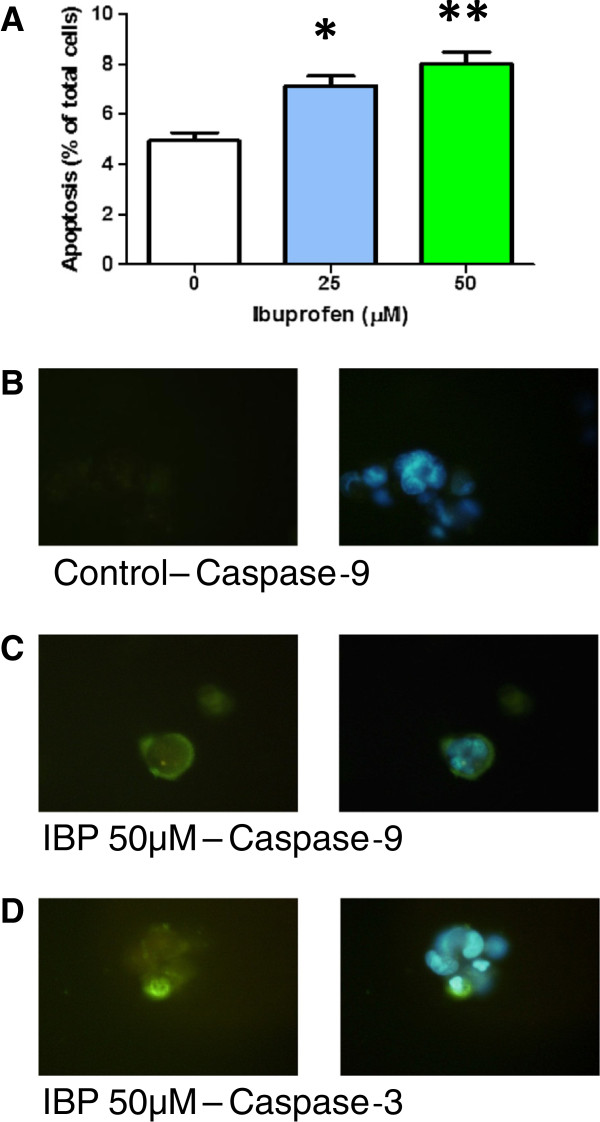
**Effect of IBP on apoptosis of T98G cells.** (**A**) Morphological changes in the nuclei typical of apoptosis were analysed after staining with Hoechst 33342. An increase in apoptosis was seen as both 25 and 50 μM. Statistical significance, *p = 0.0010 and ** p = 0.0001, n = 8. (**B**-**D**) Representative images of cleaved substrates for caspase-9 and caspase-3 in live control and 50 μM IBP treated cells. Cells with green fluorescence have activated caspase-9 or 3. Nuclei stained with Hoechst 33342.

The effects of IBP on apoptosis were analysed by Hoechst 33342 nuclear staining and visualization of caspase-9 and caspase-3 activity in live cells. Data from Hoechst 33342 (Figure 4A) showed a higher percentage of apoptotic nuclei in IBP cells compared to the control group. Apoptosis increased by 167% in the presence of 50 μM IBP. Evidence of caspase-9 and caspase-3-like activity was seen in the form of fluorescence substrate cleavage only in the 50 μM IBP treated cells and representative images are shown in Figure 4B-D.

### Effect of IBP on cell migration

**Figure 5:**
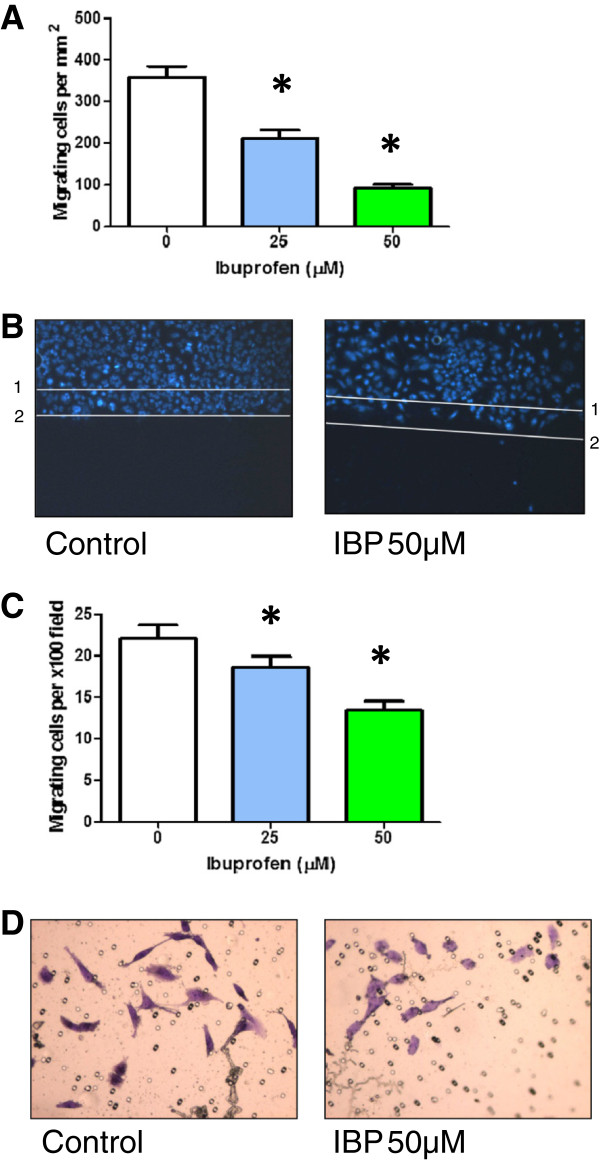
**Effect of IBP on T98G cell migration.** (**A**) Migration of T98G in the presence or absence of 25 μM or 50 μM IBP in the monolayer scratch assay. Number of migrating cells per unit area. Statistical significance, * p < 0.05 vs control, n = 8 in control, n = 16 in 25 μM and n = 16 in 50 μM. (**B**) Representative images of Hoechst 33342-labelled control and 50 μM IBP treated cells. Start point at time zero indicated by line1 and average maximum migratory distance possible in a 10 h period indicated by line 2. (**C**) Migration of T98G in the presence or absence of 25 μM or 50 μM IBP in the transwell assay. Number of migrating cells per x100 field. Statistical significance, * p < 0.0005 vs control, n = 12. (**D**) Representative images of crystal violet stained control and 50 μM IBP treated cells.

To investigate the effect of IBP on glioma cell migration ability, scratch migration assays and transwell migration assays were performed. As shown in Figure 5A-B, cell migration in the scratch migration assay was reduced by 40% in cells treated with 25 μM ibuprofen and 74% in cells treated with 50 μM IBP. Inhibition of migration was also seen in the transwell assays with 36% inhibition at 50 μM compared with control cells (Figure 5C-D).

### Effect of PGE_1_ and PGE_2_ on cell proliferation and apoptosis

**Figure 6:**
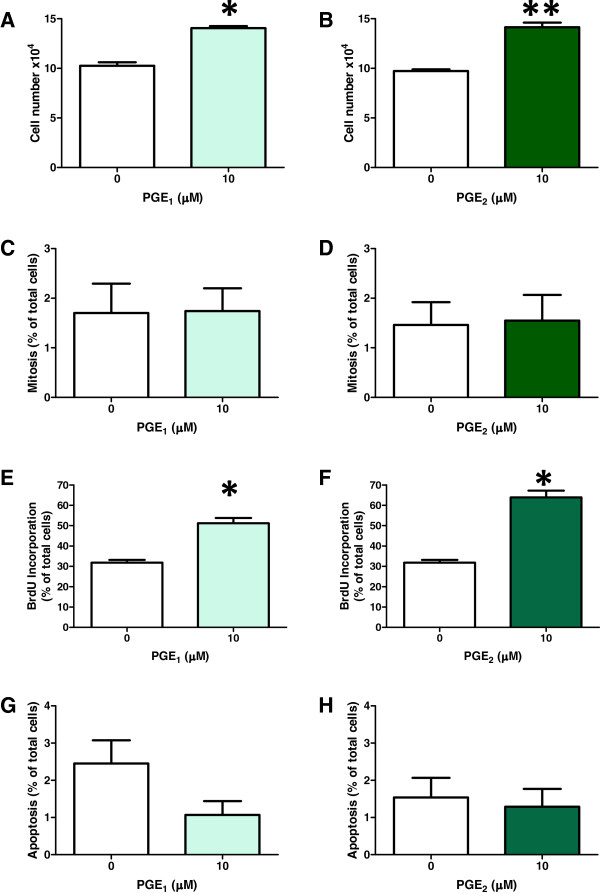
**Effect of PGE**_**1**_**and PGE**_**2**_**on cell number, mitotic index, bromodeoxyuridine (BrdU) incorporation and apoptosis in T98G cells.** (**A**) Both PGE_1_ and PGE_2_ increased cell number at 10 μM after 72 h treatment. Statistical significance *p = 0.0007 and **p = 0.0008, n = 4. (**B**) The number of cells in mitosis was obtained by Hoechst 33342 staining, and counting at x200 magnification. n = 8. (**C**) The nuclear incorporation of BrdU in the S-phase was measured after DAB staining and methyl green counterstaining. Statistical significance *p < 0.0001, n = 4. (**D**) Morphological changes in the nuclei typical of apoptosis were analysed after staining with Hoechst 33342. Each experiment was repeated at least three times.

In the case of treatment with exogenous PGE_1_ and PGE_2_, the results showed an increase of 37% with PGE_1_, while PGE_2_ increased proliferation by 45% when compared with the control (Figure 6A-B). When mitotic index was measured the presence of PGE’s did not have a significant effect (Figure 6C-D). However, the percentage of BrdU positive cells in cells treated with PGE_1_ or PGE_2_ was increased by 62% and 100%, respectively (Figure 6E-F).

The effect of PGE_1_ and PGE_2_ on apoptosis was also analysed by Hoechst 33342 nuclear staining, and detection of caspase-9 and caspase-3 activity. Data from Hoechst 33342 staining (Figure 6 G-H) showed no difference in the number of apoptotic cells. The presence of activated caspase-9 and caspase-3 in the PGE treated cells was no different from the very low levels found in the control cells, unlike the results found for IBP treated cells (data not shown).

### Effect of PGE_1_ and PGE_2_ on cell migration

**Figure 7:**
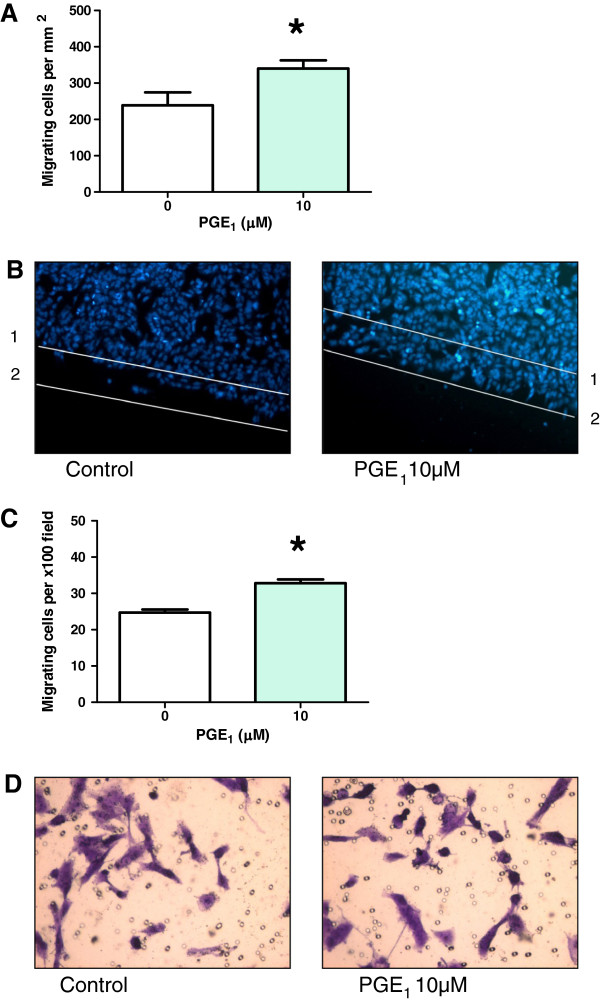
**Effect of PGE**_**1**_**on T98G cell migration.** (**A**) Migration of T98G in the presence or absence of 10 μM PGE_1_ in the monolayer scratch assay. Number of migrating cells per unit area. *p = 0.0001, n = 8 (**B**) Representative images of Hoechst 33342-labeled control and 10 μM PGE_1_ treated cells. Start point at time zero indicated by line1 and average maximum migratory distance possible in a 10 h period indicated by line 2. (**C**) Migration of T98G in the presence or absence of 10 μM PGE_1_ in the transwell assay. Number of migrating cells per x100 field. Statistical significance *p = 0.0001, n = 16. (**D**) Representative images of crystal violet stained control and 10 μM PGE_1_ treated cells.

**Figure 8:**
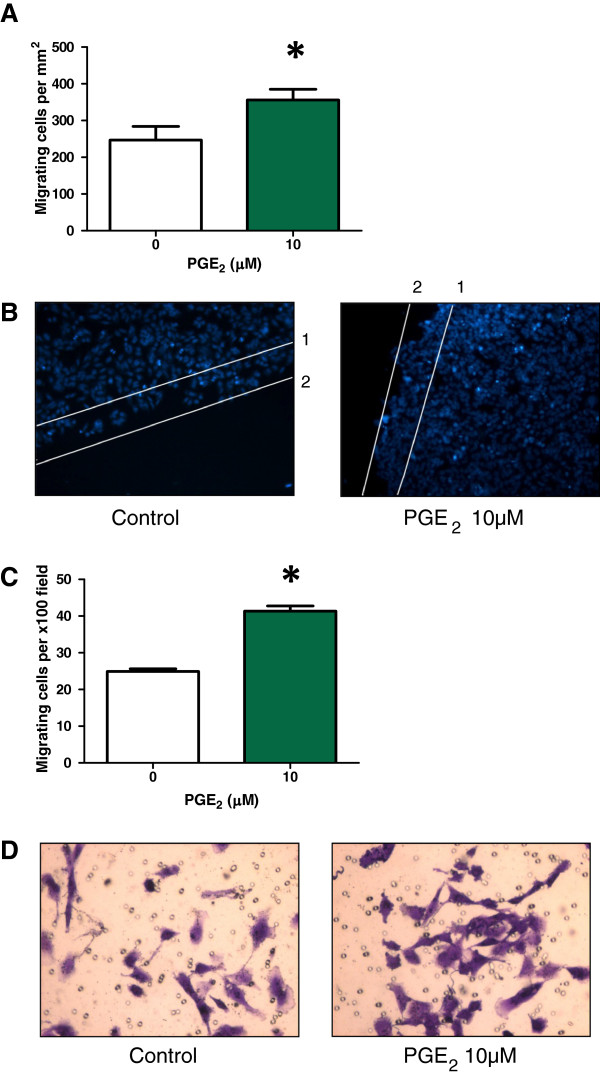
**Effect of PGE**_**2**_**on T98G cell migration.** (**A**) Migration of T98G in the presence or absence of 10 μM PGE_2_ in the monolayer scratch assay. Number of migrating cells per unit area. *p = 0.0001, n = 8 (**B**) Representative images of Hoechst 33342-labeled control and 10 μM PGE_2_ treated cells. Start point at time zero indicated by line1 and average maximum migratory distance possible in a 10 h period indicated by line 2. (**C**) Migration of T98G in the presence or absence of 10 μM PGE_2_ in the transwell assay. Number of migrating cells per x100 field. Statistical significance *p = 0.0001, n = 16. (**D**) Representative images of crystal violet stained control and 10 μM PGE_2_ treated cells.

Unlike IBP, the treatment with exogenous PGE_1_ and PGE_2_ increased cell migration in both assays. PGE_1_ increased migration by 43% in the scratch migration assay and by 28% in the transwell assay (Figure 7A-D). PGE_2_ increased migration by 44% in the scratch migration assay and by 68% in the transwell assay (Figure 8A-D).

## Discussion

Glioblastoma multiforme (GBM), a highly invasive and vascularized tumour, responds poorly to conventional cytotoxic therapy. In our study, we showed that IBP potently inhibited proliferation, migration and induced apoptosis in T98G human glioma cells. It has been shown that several NSAIDs are able to induce glioma cell apoptosis [22]. Previous studies have shown that in various tumours a high concentration of PGE_2_ and a high COX-2 activity are present. Overexpression of COX-2/PGE_2_ in human tumors is associated with progression, invasion and angiogenesis [23-25]. However, the mechanism of COX-2/ PGE_2_ up regulation in glioma cells is still poorly defined.

IBP, a generic and relatively inexpensive, non-selective COX inhibitor, is still widely used in clinical practice in the treatment of various cancers including colon and prostate cancer [3-5]. Despite enthusiasm about the potential usefulness of NSAIDs as anticancer agents, little has been reported about the effects of IBP on brain tumours. Therefore, the aim of this study was to investigate the effect of IBP upon the proliferation, migration and apoptosis processes in glioma cells.

Studies demonstrated that the proliferation and invasion of cultured glioma cell lines was inhibited in vitro by the specific COX-2 inhibitor NS-398, suggesting a functional role for COX-2/ PGE_2_ in glioma [26]. In our study we showed that IBP in doses of 25 μM and 50 μM inhibited the proliferation of T98G cells dose and time dependently. These findings are compatible with a study that showed that inhibition of COX activity and expression blocked the release of PGE_2_ from U87-MG human glioma cells and this generated a decrease in their proliferation [27]. In a recent study nano-prodrugs of IBP caused a greater reduction in cell proliferation in comparison with the nano-prodrugs of indomethacin or naproxen in the U87-MG cell line [28]. PGE_2_ plays an important role in the pathogenesis of endometriosis. Inhibition of COX-2 was recently reported to decrease migration as well as invasion of human endometrial epithelial and stromal cells. Results of the study indicate that selective inhibition of PGE_2_ receptors EP2 and EP4 suppresses expression and/or activity of matrix metalloproteinase (MMP2 and MMP9) proteins and increases expression of tissue inhibitor of metalloproteinases (TIMP1, TIMP2, TIMP3, and TIMP4) proteins and thereby decreases migration [29]. In another study PGE_2_ treatment significantly increased cell adhesion, migration, and invasion in hepatocellular carcinoma (HCC) cells. In addition, the effects of PGE_2_ were found to be associated with focal adhesion kinase (FAK) activity [30]. Indomethacin treatment reduced PGE_2_ content in normal and tumour tissue colorectal cancer. This reduction in tumour tissue PGE_2_ content was related to significant alterations in the expression of several hundred genes including genes related to cell cycle control and apoptosis [31]. Interestingly, indomethacin-loaded nanocapsules inhibited the growth of glioma cells in vitro and the C6 rat glioma model in vivo [32,33].

Our results demonstrated that T98G glioma cells showed signs of apoptosis after treatment with both concentrations of IBP. We found that after IBP treatment, chromatin condensation and fragmentation were increased in the nuclei. In a recent study, the treatment of U87-MG glioma cells with IBP nano-prodrugs (25 μM and 50 μM) resulted in a significant increase in cell death [27]. In another study the NSAID aspirin induced apoptosis by the inhibition of cyclin D1 and Bcl-2 in the A172 glioblastoma cell line [34]. From these studies it is apparent that inhibition of PG production by NSAIDs causes the induction of apoptosis in glioma cells.

In conclusion, our results demonstrated that IBP treatment suppressed T98G human glioma cell proliferation and apoptosis in vitro. In addition IBP caused a significant inhibition of T98G migration. In contrast, the addition of either exogenous PGE_1_ or PGE_2_ caused a significant increase in cell proliferation and increased cell migration. The difference in PGE_1_ and PGE_2_ effects on cell migration may be related to the expression profile of EP and IP receptors in the T98G cell line and requires further investigation. Altering prostaglandin metabolism remains a promising target for GBM treatment and is currently being studied in our laboratory.

## Competing interests

The authors declare that they have no competing interests.

## Authors’ contributions

RNG participated in the execution and analysis of the study. AC participated in the design and analysis of the study. Both authors read and approved the final manuscript.

## Funding

The study was funded by FAPESP (Fundação de Amparo à Pesquisa do Estado de São Paulo) and CAPES (Coordenação de Aperfeiçoamento de Pessoal de Nível Superior).
